# Colonization by orchid mycorrhizal fungi primes induced systemic resistance against necrotrophic pathogen

**DOI:** 10.3389/fpls.2024.1447050

**Published:** 2024-07-31

**Authors:** Galih Chersy Pujasatria, Chihiro Miura, Katsushi Yamaguchi, Shuji Shigenobu, Hironori Kaminaka

**Affiliations:** ^1^ The United Graduate School of Agricultural Sciences, Tottori University, Tottori, Japan; ^2^ Faculty of Agriculture, Tottori University, Tottori, Japan; ^3^ Functional Genomics Facility, National Institute for Basic Biology Core Research Facilities, Okazaki, Japan; ^4^ Unused Bioresource Utilization Center, Tottori University, Tottori, Japan

**Keywords:** *Bletilla striata*, defense priming, *Dickeya fangzhongdai*, induced systemic resistance (ISR), mycorrhizal fungi, necrotrophic pathogen, orchids

## Abstract

Orchids and arbuscular mycorrhiza (AM) plants evolved independently and have different structures and fungal partners, but they both facilitate nutrient uptake. Orchid mycorrhiza (OM) supports orchid seed germination, but unlike AM, its role in disease resistance of mature plants is largely unknown. Here, we examined whether OM induces systemic disease resistance against a necrotrophic pathogen in a similar fashion to AM. We investigated the priming effect of mycorrhizal fungi inoculation on resistance of a terrestrial orchid, *Bletilla striata*, to soft rot caused by *Dickeya fangzhongdai*. We found that root colonization by a compatible OM fungus primed *B. striata* seedlings and induced systemic resistance against the infection. Transcriptome analysis showed that priming was mediated by the downregulation of jasmonate and ethylene pathways and that these pathways are upregulated once infection occurs. Comparison with the reported transcriptome of AM fungus–colonized rice leaves revealed similar mechanisms in *B. striata* and in rice. These findings highlight a novel aspect of commonality between OM and AM plants in terms of induced systemic resistance.

## Highlights

- Unlike arbuscular mycorrhizal (AM) plants, our knowledge of orchid mycorrhizal fungus ability to induce systemic resistance is limited. Orchid mycorrhiza evolves independently from AM plants but retains common components for the regulation of symbiosis at molecular levels (Miura et al., Mol. Plant Microbe Interact., 2018). Thus, we are aiming to explore this commonality between the two in terms of their impact on the host plant defense response against pathogens. This study elaborates the positive correlation between *Bletilla striata* root colonization by mycorrhizal fungus and induced systemic resistance against a necrotrophic pathogen, *Dickeya fangzhongdai* (Pectobacteriaceae). The magnitude of systemic resistance is determined by the compatibility with mycorrhizal fungus itself. Also, comparative transcriptomic results shows that priming by *T. calospora* involves upregulation of jasmonate, and ethylene, which is commonly known to occur in AM plants. Our findings contribute to a better understanding of mycorrhiza contribution to induced systemic resistance in orchids as well as its molecular basis. This work is important because not only orchid mycorrhiza is understudied but also the understanding of induced systemic resistance in orchids may give clues to the mechanisms of the evolution of orchid mycorrhiza and its impact on survival against diseases.

## Introduction

Mycorrhiza is one of the oldest symbioses, in which plant roots are colonized by fungi forming a specialized structure inside or outside root cells where nutrient uptake and transfer occur (Smith and Read, 2008). The most common type of mycorrhiza, arbuscular mycorrhiza (AM), is found in almost 80% of flowering plants (Brundrett and Tedersoo, 2018) and appeared in the rooting system at the latest during the Carboniferous (Strullu-Derrien et al., 2018). During mycorrhizal colonization, AM fungi transfer nitrogen, phosphate and water to the host plants (Parniske, 2008; Kakouridis et al., 2022). In return, plants provide carbon compounds to the fungi. The extent of each of these physiological functions often differs among mycorrhizal types and plant species. On the basis of these differences, several other mycorrhizal types are distinguished, including ectomycorrhiza, orchid mycorrhiza (OM), and ericoid mycorrhiza. OM is unique in that the fungal structures formed inside orchid cells are transient and are gradually degraded; this phenomenon is most prominent during early seed germination (Selosse et al., 2017). Mycorrhizal associations in orchids are obligatory during early germination, although in green, leafy species their extent decreases with time due to the plants’ autotrophic nature (Cameron et al., 2008; Girlanda et al., 2011; Wang et al., 2023).

Orchidaceae have evolved endomycorrhizas with Basidiomycota or occasionally Ascomycota (Fracchia et al., 2016). The dominant group, *Rhizoctonia-*like fungi, includes three main genera—*Ceratobasidium*, *Sebacina/Serendipita*, and *Tulasnella* (Smith and Read, 2008)—while some lower taxa of subfamily Epidendroideae are sometimes associated with ectomycorrhizal fungi (Bidartondo et al., 2004; McKendrick et al., 2004; Zahn et al., 2023) or even wood-rotting, mushroom-forming fungi (Umata et al., 2013). Both OM and AM are involved in nitrogen, carbon, and phosphate transfer (Cameron et al., 2007, 2008; Kuga et al., 2014; Fochi et al., 2016) and have common integral molecular mechanisms (Miura et al., 2018). It was recently discovered that gibberellin inhibits both OM and AM colonization, but it inhibits seed germination only in orchids (Miura et al., 2024). These findings further encourage the exploration of the commonalities between OM and AM, including ecophysiological aspects such as disease resistance.

Two distinct defense responses occur upon pathogen infection: an early, local response at the infection site and a systemic response at distal sites (David et al., 2019); the latter includes systemic acquired resistance (SAR) (Ross, 1966) and induced systemic resistance (ISR) (van Peer et al., 1990; Wei et al., 1991). Systemic acquired resistance occurs when pathogens directly infect leaves and involves mainly salicylic acid, whereas ISR is induced by beneficial microbes (bacteria, endophytic fungi, and mycorrhiza) interacting with roots and involves jasmonate and ethylene (Vlot et al., 2021). During ISR, the plant enters an alert state called priming, allowing enhanced resistance once infection occurs, mainly against necrotrophic pathogens (Pieterse et al., 2014). Primed plants often show no visible changes before infection; thus, ISR is best studied in plants challenged with pathogens, which allows changes at the cellular level between non-primed and primed plants to be compared. Due to the diversity of microbes interacting with roots, the molecular mechanisms of ISR are more diverse than those of SAR because different microbe species induce the accumulation of different signaling compounds (Haney et al., 2018; Dreischhoff et al., 2020). ISR induced by an AM fungus in *Medicago truncatula* increases the defense response against *Xanthomonas campestris* (Liu et al., 2007), whereas *Solanum lycopersicum* has a similar response against *Fusarium oxysporum* (Wang et al., 2022a). Almost all types of pathogens attack orchids, including bacteria (Keith et al., 2005; Suharjo et al., 2014; Khamtham and Akarapisan, 2019), fungi (Silva and Pereira, 2007; Lopes et al., 2009; Srivastava et al., 2018; Suwannarach et al., 2018), and viruses (Fogell et al., 2019; Tsai et al., 2022). Very little is known about ISR in orchids; to the best of our knowledge, only two studies have reported the occurrence of ISR and its role in alleviating soft rot (Wu et al., 2011; Ye et al., 2019). Thus, enhancing our knowledge on ISR in orchids will give clues on the consequences of evolving mycorrhizal association.

On the basis of the arguments that colonization by AM fungi primes host defense responses against pathogens (Marquez et al., 2018) and major molecular components of AM symbiosis signaling are also present in OM (Miura et al., 2018), here we hypothesized that colonization by OM fungi (OMF) also causes ISR in orchids just as in AM plants. We used a necrotrophic, pectinolytic Gram-negative bacterium known to cause leaf soft rot in orchids (Cating and Palmateer, 2011; Joko et al., 2014; Wei et al., 2021). As recommended by Eck and co-workers (Eck et al., 2022), we also investigated the relationship between mycorrhizal colonization rate and ISR. We focused on a generalist orchid species, *Bletilla striata* (Orchidaceae, tribe Arethuseae), because it is relatively easy to grow and it associates with OMF from different genera (Zettler et al., 2001; Yamamoto et al., 2017; Miura et al., 2019). Using two OMF species previously reported to associate with *B. striata* (Miura et al., 2019; Fuji et al., 2020) we evaluated ISR and its molecular regulation in leaves through transcriptomic analysis.

## Materials and methods

### Plant and fungal materials

*Bletilla striata* Rchb.f. ‘Murasakishikibu’ plants were purchased from a local nursery in Japan (Yamamoto et al., 2017). For seed production, flowers were self-pollinated and allowed to set seeds for 6 months (until dehiscence). Seeds were randomly picked from capsules harvested between 2017 and 2020. In a pathogen inoculation assay, aside from *B. striata*, *Arabidopsis thaliana* (L.) Heynh. ecotype Columbia (Col-0) and *Nicotiana benthamiana* were also used.

Our earlier studies showed that several strains of *Tulasnella* and *Serendipita vermifera* form OM associations with *B. striata* during seed germination (Yamamoto et al., 2017; Miura et al., 2019; Fuji et al., 2020). Among those fungi, we chose *T. calospora* (Boud.) Juel MAFF305805 (coded as T.cal05) and *S. vermifera* (Oberw.) P. Roberts MAFF305830 (coded as S.ver30).

### Symbiotic seed germination

In symbiotic seed germination, *S. vermifera* and *T. calospora* were cultured in agar (1.5 g/L) containing 2.5 g/L oatmeal (Becton Dickinson, Sparks, MD, USA) until maximum hyphal growth. *Bletilla striata* seeds were surface-sterilized in 3 mL of 5% NaOCl containing 5 µL Tween-80 for 2 min and were sown directly on top of the mycelia. The plates were incubated at 25°C for 2 weeks.

### Asymbiotic seed germination and direct root inoculation of OMF

In asymbiotic seed germination, *B. striata* seeds were surface-sterilized as above and sown on solid half-strength Murashige–Skoog (MS) medium supplemented with 20 g/L sucrose, 20 g/L banana homogenate, 1 g/L tryptone, 3 g/L activated charcoal, MS vitamin mixture (pH 5.8), and 0.8 g/mL agar. Seedlings were grown for 8 weeks until formation of true leaves and roots. For the first subculture, the plantlets were transferred into half-strength P668 medium (Phytotech Labs, Lenexa, KS, USA) supplemented with 20 g/L sucrose, 0.1 g/L tryptone, 0.2 mg/L naphthalene acetic acid, 0.5 mg/L 6-benzylaminopurine, MS vitamin mixture (pH 5.8), and 0.8 g/mL agar for another 6–8 weeks. Seedlings with at least two true leaves and new roots longer than 2 cm were transferred into small pots filled with a mixture of *akadama* soil, *kanuma* soil, and vermiculite (4:4:1 volume ratio) under a 16 h light/8 h dark photoperiod at 25°C for another 2 weeks for hardening (**Supplementary Figure 1**). *S. vermifera* and *T. calospora* were cultured in YEPG liquid medium containing 3 g/L yeast extract, 3 g/L peptone, and 20 g/L glucose for 4 weeks until sufficient mycelial growth. The mycelial mass was harvested and homogenized in distilled water (4 mL/g mycelial mass) using a homogenizer (Nissei, Osaka, Japan). A 2-mL aliquot of the mycelial suspension was inoculated beneath each seedling.

### Visualization and quantification of OMF colonization in protocorms and roots

Root colonization is defined as the presence of pelotons, both intact and degraded (Yamamoto et al., 2017). Protocorms and roots were fixed in 70% ethanol and cleared in 5% KOH at 90°C for 1 h (Peterson et al., 2004). To evaluate colonization, the protocorms were stained with 5% black ink (Sheaffer, Fort Madison, IA, USA) in 5% acetic acid for 10 min (Vierheilig et al., 1998). Protocorms were photographed under a light microscope (BX53; Olympus, Tokyo, Japan) equipped with a digital camera (DP27; Olympus). By using ImageJ v.1.53a, pelotons present in protocorms were counted with the multipoint tool and root colonization was measured with the segmented line tool. Root colonization was quantified as described: each piece of root was divided into imaginary 1-mm segments. Each segment was further divided horizontally into four subsegments, especially the middle part by the vascular bundle. The ratio of cortical colonization within each segment was scored as 0 (no OMF colonization on either side), 0.25 (one-quarter of the segment is colonized), 0.5 (only one side is fully colonized), 0.75 (three-quarters of the segment is colonized), or 1 (both sides are colonized). The colonization rate (CR) for a single root was calculated through the colonization index (CI) using the following equations:


$$CR=CI\times 100\%\;with\;CI=\frac{0.25\sum^{\;}n_{0.25}+0.5\sum^{\;}n_{0.5}+0.75\sum^{\;}n_{0.75}+\sum^{\;}n_{1}}{\sum^{\;}n_{0}+\sum^{\;}n_{0.25}+\sum^{\;}n_{0.5}+\sum^{\;}n_{0.75}+\sum^{\;}n_{1}}\;,$$

where *i* is a positive integer from 0 to 4, *n_i/_*_4_ is the number of segments colonized at a particular level, and *N* is the total number of segments in one root. This index can vary from 0 to 1. The final CR of a seedling was then expressed as the arithmetic average value of CR obtained from all roots. The detailed method is available at Zenodo (https://doi.org/10.5281/zenodo.10464582).

### Pathogen inoculation

*Erwinia chrysanthemi* MAFF311045, which was isolated from *Phalaenopsis* (Suharjo et al., 2014), was cultured in Luria–Bertani (LB) medium overnight. Bacterial suspension at several concentrations and OD_600_ = 0.0025 was mixed with 0.01% Tween-20 (10:1 volume ratio). Two leaves per seedling of *B. striata*, *A. thaliana*, and *N. benthamiana* were used. For each leaf, one small drop (3 µL) was placed on the syringe-wounded adaxial surface. During infection, high humidity was maintained by flooding the planting tray with water and covering the plantlets with a moist plastic lid. Symptoms were observed and leaf samples were collected 3 days after inoculation. Infected leaves (diameter 4 mm) were excised at the site of initial infection and macerated in 1 mL liquid selective enhancement medium containing 3.75 g/L MgSO_4_·7H_2_O, 1 g/L (NH_4_)_2_SO_4_, 1 g/L K_2_HPO_4_, 0.2 mL/L 5N NaOH, and 1.7 g/L pectin at pH 7.2 (Wako Pure Chemicals, Osaka, Japan) for 24 h at 28°C (Hélias et al., 2012). Because of the ability of this pathogen to produce indigoidine (Lee and Yu, 2006; Alič et al., 2019; Wei et al., 2021), we used a selective nutrient agar medium containing crystal violet, 70 mg/mL glutamine, and 4 mg/mL MnCl_2_·2H_2_O at pH 6.5–6.8. Plates were incubated at 28°C for 1 day and bluish colonies were selected for counting.

### Leaf symptoms, indicators of photosynthetic damage, and peroxide content

To visualize the necrotic area, leaves were immediately placed into 0.05% trypan blue, stained at 37°C overnight, and destained in absolute ethanol for another night. To visualize peroxide distribution around the infected area, infected leaf samples were directly incubated in 1 mg/mL diaminobenzidine at 37°C overnight in the dark. The leaves were fully decolorized in several changes of 70% ethanol at 70°C for 1 h and stored in 30% glycerol until observation.

Photosynthetic quantum yield was measured with a miniPPM-300 photosynthesis meter (EARS, Wageningen, Netherlands) after dark preconditioning for at least 30 min. Subsequently, individual leaves were weighed, and chlorophyll was extracted with 1 mL of dimethyl sulfoxide per leaf overnight. Quantification of total chlorophyll content was based on extract absorbance at 649 and 665 nm and an equation suggested by (Wellburn, 1994). The values expressed in µg/mL solvent were converted into µg/g leaf fresh weight.

The total peroxide content of leaves was measured by luminol chemiluminescence catalyzed by Co^2+^ (Pérez and Rubio, 2006). Samples were frozen in liquid nitrogen, ground in 0.5 mL 5% trichloroacetic acid, and centrifuged at 13,000 rpm and 4°C for 10 min. The supernatant was supplemented with 5% polyvinylpyrrolidone and diluted 100-fold with water. An aliquot of the diluted extract (20 µL) was added to 1 mL of diluted luminol–cobalt mixture (1 g/L luminol and 0.7 g/L CoCl_2_·6H_2_O in carbonate buffer pH 10.2, incubated for at least 1 h, and diluted 10-fold before use). Chemiluminescence was measured 5 s after the addition of luminol in a Gene Light GL-220 luminometer (Microtec Nition, Chiba, Japan). The values were expressed as log_10_ (relative light unit)/g fresh weight.

### RNA extraction and RNA-sequencing

Aside from control, only *T. calospora-*colonized seedlings, regardless of their infection status, were used for RNA extraction and sequencing. Each replicate contained materials collected from at least five individuals. Total leaf RNA was extracted using a Total RNA Extraction Kit (Plant) (RBC Bioscience, Taipei, Taiwan) according to the manufacturer’s protocol. Residual genomic DNA was removed with RNase-free DNase I (Toyobo, Osaka, Japan). The quality and quantity of the total RNA were checked using a Qubit RNA HS Assay Kit and Qubit 2.0 Fluorometer (Thermo Fisher Scientific, Waltham, MA, USA). RNA-seq library construction from the total RNA using an MGIEasy RNA Directional Library Prep Set (MGI, Shenzhen, China) and sequencing with strand-specific and paired-end reads (150 bp) using the DNBSEQ-T7RS sequencing platform were performed by Genome-Lead Co. (Takamatsu, Kagawa, Japan).

### Transcriptome analysis

The obtained raw reads were filtered using Fastp v.0.23.2 (Chen et al., 2018) to remove low-quality reads (<Q30) and adapter sequences. Reads were mapped to *de novo* assembled transcript sequences (Miura et al., 2018) using Bowtie2 v.2.5.2 (Langmead and Salzberg, 2012) (**Supplementary Table 1**). Differentially expressed genes (DEGs) were analyzed using the *edgeR* package. Library size was corrected using the trimmed mean of M value method and fitted using the *glmTreat* (filtered at logFC 1.5) (Smyth et al., 2018). Gene Ontology (GO) enrichment was analyzed in the *topGO* package (Alexa and Rahnenfuhrer, 2023) using the *elim* parameter and Fisher exact test, and the *p*-values were adjusted with the Benjamini–Hochberg method. The false discovery rate (FDR) threshold was set at 0.05. Genes of *B. striata* orthologous to rice genes were identified using SonicParanoid (Cosentino and Iwasaki, 2019) with default parameters. The rice genome assembly (Os-Nipponbare-Reference-IRGSP-1.0; Kawahara et al., 2013) were retrieved from Ensembl Plants (https://plants.ensembl.org/index.html). Additional genes involved in jasmonate, ethylene biosynthesis, and phosphate metabolism were retrieved from rice transcriptomic data (Campo and San Segundo, 2020) and used as queries for local TBLASTX against *B. striata* genome assembly. Only *B. striata* genes with an E-value less than 10^−5^ were selected. The whole TBLASTX process was conducted in Genetyx v.15 software (Genetyx, Tokyo, Japan). For visualization, a heatmap was generated in the RStudio v.4.1.2 package *Complexheatmap* (Gu et al., 2016), and an UpSet plot was generated manually using CorelDRAW 2021 v.23.1.0.389 (Alludo, Ottawa, ON, Canada).

### Statistical analysis

For protocorm symbiotic cell count, root colonization rate, *D. fangzhongdai* titer, photosynthetic quantum yield, chlorophyll content, and peroxide content data, whenever the data did not meet the normality assumption, logarithmic data transformation was used to enable analyses with parametric tests according to the corresponding data skewness. The statistical analyses were conducted in RStudio v.4.1.2 with Student’s *t-*test, ANOVA, or a Kruskal–Wallis test. To investigate whether the relationship between *D. fangzhongdai* titer and root colonization rate among individual seedlings with leaf soft rot is linked to OMF identity, we used a generalized linear mixed model (GLMM) for negative binomial implemented in the *lmerTest* package (Kuznetsova et al., 2017). Considering the possibility of different responses among individuals and infection occurrence, experimental batches and individual seedlings were used as a random factor and OMF identity and colonization rate were used as fixed factors. The final syntax was constructed without the interaction between OMF colonization rate and identity to produce, the most maximal yet parsimonious model: *glmer.nb* (colony-forming unit [CFU] count ~ colonization rate + OMF identity + [1| experimental batch/treatment]). To determine the importance of OMF colonization rate and OMF identity in determining CFU count, the *model.sel* function of the *MuMIn* package (Bartoń, 2023) was applied to the constructed GLMM. The omitted factor with the largest change in the corrected Akaike information criterion value was regarded as the most important fixed factor.

## Results

### *Tulasnella calospora* and *S. vermifera* colonize *B. striata* roots with similar symbiotic affinity in protocorms

**Figure 1:**
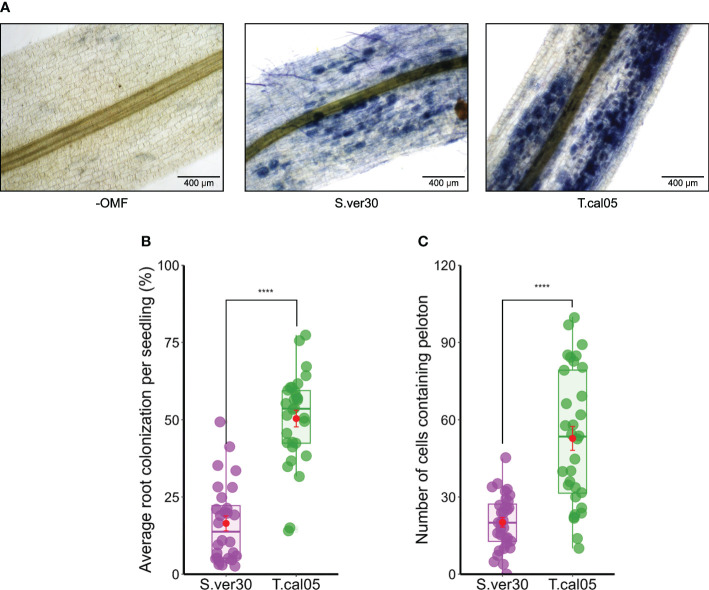
Colonization of *Bletilla striata* by orchid mycorrhizal fungi (OMF). Seedlings were inoculated with the homogenized mycelial suspension of OMF into soil and incubated for 2 weeks. **(A)** Root colonization of OMF was visualized by ink staining. Pelotons are visible inside the root cortex as blue dots. Bar = 400 µm. **(B)** Average root colonization rate per individual seedling. Symbiotic seed germination was conducted by co-culture with OMF for 2 weeks. **(C)** Protocorms were subjected to ink staining, and the number of symbiotic cells per protocorm was counted. Red dots indicate mean ± standard error. ****p< 0.0001, Student’s *t*-test. S.ver30, *Serendipita vermifera*; T.cal05, *Tulasnella calospora*.

We established an *ex vitro* growing system for *B. striata* seedlings in a common potting mix and cultured OMF in liquid medium to obtain maximum mycelial growth and facilitate direct root inoculation. We tested the compatibility of *B. striata* with OMF candidates by direct root inoculation described in the current study, and by a symbiotic germination assay established previously (Fuji et al., 2020). Within 2 weeks, we confirmed the presence of pelotons in protocorms and roots, which is the primary sign of OM establishment (**Figure 1A**, **Supplementary Figure 2**). In protocorms, OMF colonization started from the basal part and was restricted to the lower half until the formation of shoot primordium (**Supplementary Figure 2**), as reported earlier (Miura et al., 2019). In roots, OMF colonization occurred everywhere, except the quiescent center. The colonization rate was higher for *T. calospora* than for *S. vermifera* in protocorms (**Figure 1B**) and in roots (**Figure 1C**).

### Erwinia chrysanthemi MAFF311045 is synonymous to *Dickeya fangzhongdai*

Our pathosystem for *B. striata* was based on a study on orchid infection by *Erwinia* species (Wu et al., 2011; Ye et al., 2019). We chose *Erwinia chrysanthemi* because of its ability to infect orchids. The *Erwinia* genus is rather complex (Samson et al., 2005). Our phylogenetic tree based on 16S ribosomal RNA sequences of various species within Pectobacteriaceae assigned the *E. chrysanthemi* MAFF311045 to *Dickeya fangzhongdai* (**Supplementary Figure 3**). Leaf inoculation with this pathogen resulted in a lesion and bacterial spread throughout the leaf, often causing the leaf to drop, in *B. striata*, *A. thaliana*, and *N. benthamiana* (**Supplementary Figures 4A–C**).

### Induced systemic resistance is linked to the degree of OMF colonization

**Figure 2:**
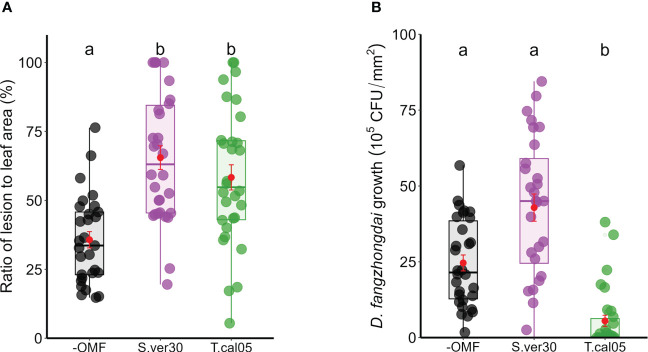
Induced systemic resistance on *Bletilla striata* leaves upon *Dickeya fangzhongdai* infection. Leaves of OMF-colonized *B striata* seedlings were inoculated with bacterial suspension of *D fangzhongdai*, and **(A)** ratio of lesion area to total leaf area and **(B)** colony-forming unit (CFU) count were measured 3 days after inoculation. Red dots indicate mean ± standard error. The different letters show that the values are significantly different at p< 0.05 based on Kruskal–Wallis test. −OMF, non-colonized control; S.ver30, *Serendipita vermifera*; T.cal05, *Tulasnella calospora*.

The effects of OMF colonization on ISR against pathogens were examined in the *B. striata*–*D. fangzhongdai* pathosystem. Both *S. vermifera* and *T. calospora* were able to colonize *B. striata* roots (**Figure 1**). Upon *D. fangzhongdai* infection, lesions on detached leaves were wider in the presence of OMF colonization than in its absence (**Figure 2A**). *T. calospora* colonization induced the defense response, as indicated by a reduced *D. fangzhongdai* titer (**Figure 2B**). On the other hand, *S. vermifera* colonization did not affect resistance since *D. fangzhongdai* titer was not reduced as in uncolonized seedlings.

**Figure 3:**
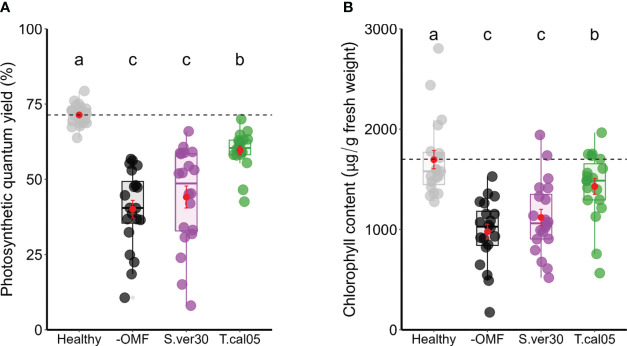
Photosynthetic damage caused by *Dickeya fangzhongdai* infection. **(A)** Photosynthetic quantum yield and **(B)** total chlorophyll contents were measured in *D fangzhongdai-*infected leaves of OMF-colonized *Bletilla striata* seedlings 3 days after infection and those of healthy uninoculated seedlings. Red dots indicate mean ± standard error. The different letters show that the values are significantly different at p< 0.05 based on Kruskal–Wallis test. −OMF, non-colonized control; S.ver30, *Serendipita vermifera*; T.cal05, *Tulasnella calospora*.

To further test the physiological changes associated with the induction of disease resistance, we assessed the indicators of photosynthetic damage and peroxide production. Photosynthetic damage is a general proxy for disease resistance in leaves, and reactive oxygen species are general inducers of disease resistance (Lu and Yao, 2018; Li and Kim, 2022). Regardless of lesion size, the necrotic area was relatively constant, as revealed by trypan blue staining (**Supplementary Figures 4D, E**). The photosynthetic quantum yield was reduced in all infected leaves, but the photosynthetic damage caused by the infection was significantly smaller when *T. calospora* colonized the roots (**Figure 3A**). Similarly, the decrease in total chlorophyll content of seedlings caused by *D. fangzhongdai* infection was lowest in the presence of *T. calospora* (**Figure 3B**). Hydrogen peroxide was also accumulated in infected leaves (**Supplementary Figure 5A**), and its content was significantly higher in OMF-colonized seedlings (**Supplementary Figure 5B**). Overall, the ability of OMF to systemically induce disease resistance in orchids is not necessarily related to symptom appearance, e.g., lesion size, but rather to reduced pathogen proliferation. This is also supported by the data that both OMF identity and colonization rate strongly affected ISR (*n* = 88 plants; *p<* 0.001; **Tables 1**, **2**, **Supplementary Figure 6**).

**Table 1:** Results of generalized linear mixed model.

Fixed effects	Estimate	Standard error	z-value	p-value
(Intercept)	0.666351	0.215476	3.092	**< 0.01**
Colonization rate	-0.020733	0.004376	-4.738	**< 0.001**
OMF identity	0.035266	0.00508	6.943	**< 0.001**

*Significances are highlighted in bold.

*Dickeya fangzhongdai* titer in leaves is used as the dependent variable.

**Table 2:** Results of model comparison using corrected Akaike Information Criterion values.

Model	Log likelihood	AICc	delta
Initial	-156.618	326.3	0
Without “colonization rate”	-170.078	350.9	24.62
Without “OMF identity”	-181.193	373.1	46.84

*Dickeya fangzhongdai* titer in leaves is used as the dependent variable.

### Transcriptome analysis of leaves of *B. striata* seedlings inoculated with OMF and pathogen

The lowest *D. fangzhongdai* titer and photosynthetic damage in *T. calospora–*infected leaves led us to hypothesize that *T. calospora* colonization enhances disease resistance by systemically inducing changes in gene transcription. We analyzed transcriptomes of leaves upon root inoculation with *T. calospora* (T treatment), upon leaf infection by *D. fangzhongdai* (P treatment), or both (PT treatment).

**Figure 4:**
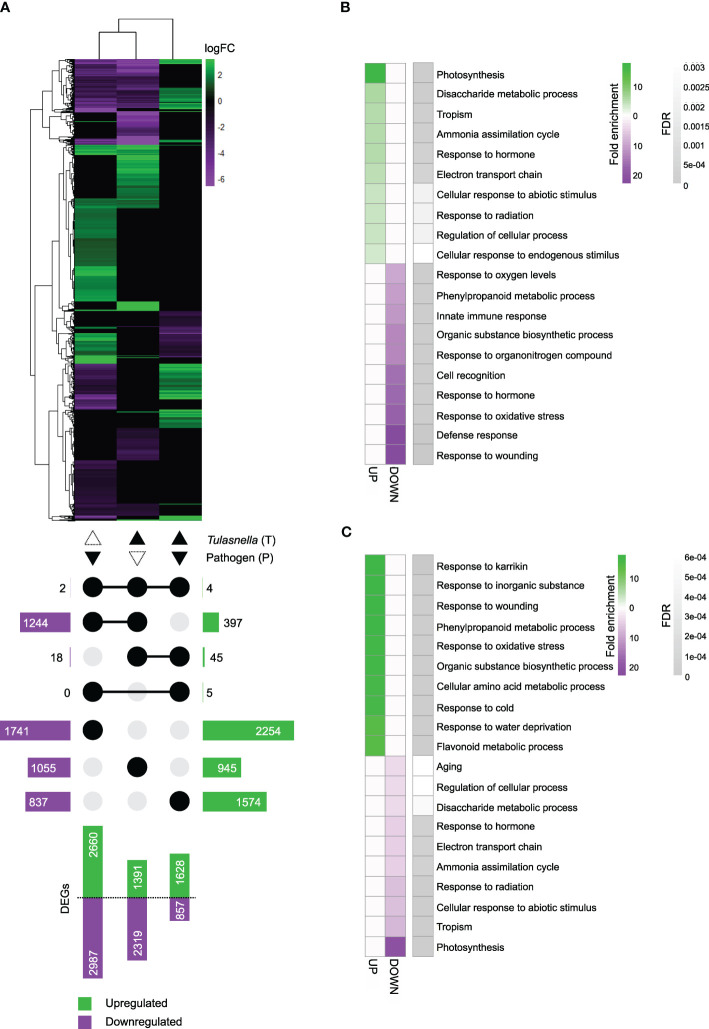
RNA-seq analysis. Root colonization of *Tulasnella calospora* in *Bletilla striata* seedlings was conducted by inoculating the homogenized mycelial suspension into soil, and then seedlings were cultured for 2 weeks. Leaves of seedlings were infected with *Dickeya fangzhongdai* by drop inoculation and harvested 3 days after inoculation. Three biological replicates (each replicate contains leaves collected from different plants) were used for RNA extraction and sequencing. **(A)** Heatmap showing fold changes of differentially expressed genes (DEGs) in leaves of seedlings treated with the pathogen *D. fangzhongdai* only (P); *T. calospora* only (T); or both (PT), each compared with non-colonized and uninoculated healthy control (without *T. calospora* and *D. fangzhongdai*). An UpSet plot below the heatmap shows the number of upregulated and downregulated DEGs attached to bar plots. Connected dots show the treatment sets that share the respective DEGs. The log fold change (logFC) was calculated as log2[treatment/uncolonized] > |1.5| with FDR< 0.05. **(B, C)** Heatmaps of the top 10 enriched biological process gene ontology (GO) terms for upregulated and downregulated DEGs in **(B)** T treatment and **(C)** PT treatment. FDR, false discovery rate. All GO terms are listed in **Supplementary Tables 5, 6**.

Up- and downregulated DEGs were identified by comparisons among the three treatments (**Supplementary Tables 2–4**). A heatmap of DEG clustering showed distinct expression patterns among the treatments (**Figure 4A**). A total of 2660 genes in P, 1391 in T, and 1628 in PT seedlings were upregulated, and 2987 in P, 2319 in T, and 857 in PT seedlings were downregulated. Among them, 6 DEGs (4 upregulated and 2 downregulated) were shared among all treatments, 1641 DEGs (397 upregulated and 1244 downregulated) between P and T, 63 DEGs (45 upregulated and 18 downregulated) between T and PT, and 5 upregulated DEGs between P and PT (**Figure 4A**). The transcriptional profile was less similar between T and PT seedlings than between P and PT seedlings, as indicated by MDS plot (**Supplementary Figure 7**). These data demonstrated that OM symbiosis greatly affects the leaf transcriptome. Since both T and PT treatments involved *T. calospora* colonization, it systemically and conspicuously altered leaf transcriptional changes before and after *D. fangzhongdai* infection.

GO enrichment analysis of biological processes revealed that terms related to photosynthesis were overrepresented among the upregulated DEGs in T-treated seedling leaves. The categories of disaccharide metabolic process, tropism, ammonia assimilation cycle, and innate immune response were also enriched. Among downregulated DEGs, response to wounding, defense response, and response to oxidative stress were among the overrepresented terms (**Figure 4B**, **Supplementary Table 5**). In PT-treated seedlings, terms related to responses to karrikin, inorganic substance, and wounding were overrepresented among the upregulated DEGs, whereas photosynthesis, tropism, and ammonia assimilation cycle were among the overrepresented terms among downregulated DEGs (**Figure 4C**, **Supplementary Table 6**). These results implied that upon *T. calospora* colonization probably by increasing primary metabolism, e.g., photosynthesis and sugar metabolism, and then resistance was increased upon pathogen infection. Thus, it is worth noting that there is a potential of growth-defense tradeoff.

### Comparisons of expression levels of phosphate and jasmonate/ethylene metabolism-related genes in rice and *B. striata*

**Figure 5:**
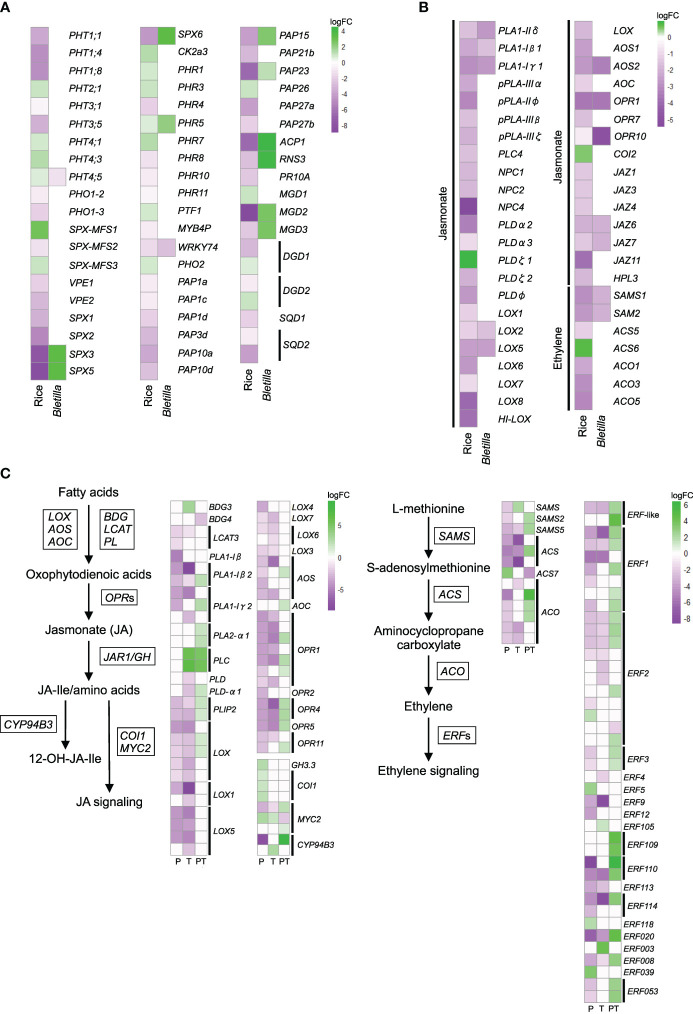
Comparative analysis of the expression patterns of phosphate-acquisition and defense hormone–related genes in leaves of arbuscular mycorrhizal rice (*Oryza sativa* spp. *japonica*, cv. Loto; Campo and San Segundo, 2020) and *Bletilla striata* seedlings treated with the pathogen *Dickeya fangzhongdai* only (P); *Tulasnella calospora* only (T); or both (PT). **(A)** Heatmap showing fold changes of DEGs involved in phosphate metabolism and **(B)** jasmonate and ethylene signaling/metabolism in T. The orthologous genes of *B striata* were identified by SonicParanoid and TBLASTX. **(C)** The expression of jasmonate- and ethylene-related genes of *B striata was* determined from transcriptomic analysis. The log fold change (logFC) was calculated as log_2_[treatment/uncolonized] > |1.5| with FDR< 0.05. See **Supplementary Tables 8, 9** for details.

We hypothesized that transcriptional patterns are conserved across different mycorrhizal types, especially in comparison with those in AM plants. To test this hypothesis, we compared our data with the published data on leaf transcriptional changes in rice (*Oryza sativa* ssp. *japonica* cv. ‘Loto’) inoculated with AM fungus *Funneliformis mosseae* (Campo and San Segundo, 2020). The most noticeable transcriptional changes in rice leaves were in phosphate- and jasmonate/ethylene metabolism-related genes. Jasmonate and ethylene are involved in resistance against necrotrophic pathogens and in ISR (Shoresh et al., 2005; Zhu, 2014). To identify the orthologous genes by SonicParanoid, we used rice genes as queries against *B. striata de novo* assembly (**Supplementary Table 7**). We also used local TBLASTX (**Supplementary Table 8**). The expression of the identified *B. striata* orthologs of phosphate metabolism–related genes did not necessarily mirror changes in the expression of rice genes (**Figure 5A**, **Supplementary Table 8**), but the identified genes involved in the first steps of jasmonate and ethylene biosynthesis were similarly downregulated in rice and *B. striata*, such as phospholipase A1 (*PLA1*), lipoxygenase (*LOX*), allene oxide synthase (*AOS*), oxophytodienoic acid reductase (*OPR*), and S-adenosylmethionine synthase (*SAMS*) (**Figure 5B**, **Supplementary Table 8**). The expression of these genes was suppressed in T and P seedlings, but increased in PT seedlings.

We further checked the expression patterns of homologous jasmonate and ethylene biosynthesis–related genes extracted from DEG lists (FDR< 0.05) and obtained similar results. The overall results showed that priming suppresses the jasmonate and ethylene signaling pathways and that leaf infection by pathogen activates them, confirming the occurrence of ISR in *B. striata* (**Figure 5C**, **Supplementary Table 9**).

## Discussion

**Figure 6:**
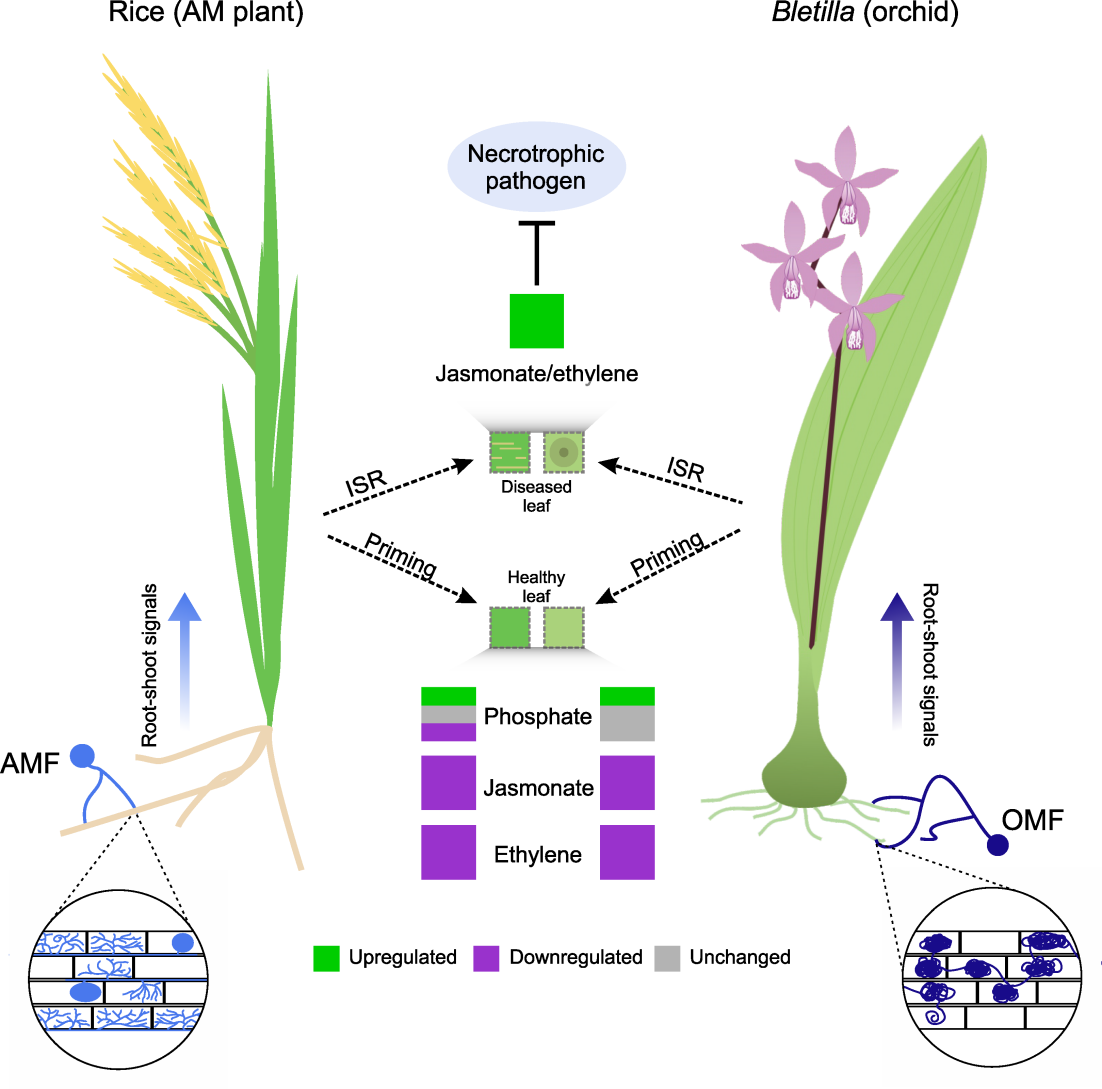
Commonalities in induced systemic resistance (ISR) primed by mycorrhizal fungus colonization in an AM plant (rice) and orchid (*Bletilla striata*). Despite structural differences, colonization by AMF and OMF causes priming by regulating the expression of genes related to primary metabolism (e.g., phosphate metabolism). This is more variable in AM plants than in orchids. However, the expression of genes related to the jasmonate and ethylene signaling pathways is downregulated in both species. When a necrotrophic pathogen infects the plants, AMF or OMF colonization triggers ISR in leaves, which increases the expression of genes involved in jasmonate and ethylene signaling pathways and in turn inhibits pathogen proliferation.

In this study, we demonstrated that a compatible OMF colonization, *T. calospora*, systemically induces resistance against a necrotrophic pathogen as well as its possible role in defense priming in orchids (**Figure 6**). We also showed partial commonalities between AM and OM plants in aboveground transcriptomic changes during root colonization.

Mycorrhizal fungi improve host fitness, which varies among AM fungus–plant pairings (Säle et al., 2021; Cope et al., 2022; Guigard et al., 2023). The driving factors for the functional diversity are largely unknown, but plants may simply choose mycorrhizal fungi that provide the highest growth with minimal metabolic costs, which is important for survival. The same applies to orchids, which underwent rapid diversification in their growth habits during the Cenozoic (Dearnaley et al., 2012; Chomicki et al., 2015; Xing et al., 2019). Orchids with the highest demand for mycorrhizal association during early seed germination prioritize different OMF species as their partners (Otero et al., 2005; Meng et al., 2019; Pujasatria et al., 2022). Previously, we showed that *B. striata* associates with multiple strains of *Tulasnella* during seed germination (Yamamoto et al., 2017; Fuji et al., 2020). It could be assumed that *B. striata* remains compatible with an OMF from seed germination until seedling development and quantified OMF colonization in seedlings with well-developed roots. Colonization during seed germination and root colonization was higher for *T. calospora* than for *S. vermifera.* Other *Bletilla* sister taxa, the members of tribe Arethuseae (e.g., *Arundina, Pleione*, and *Coelogyne*), are also associated with *Tulasnella*, as suggested by metagenomic studies and *in vitro* assays (Yukawa et al., 2009; Sathiyadash et al., 2014; Meng et al., 2019; Qin et al., 2019). These suggest that the convergent evolution of OM still involves mycorrhizal partner selection, in addition to molecular-level mechanisms shared with AM plants, such as the roles of phytohormones and transcriptional regulations (Miura et al., 2018; Miura et al., 2024).

*Dickeya fangzhongdai*, as well as *Erwinia sensu lato*, infects orchids (Fu et al., 2012; Alič et al., 2019; Ye et al., 2019; Zhou et al., 2021), *Arabidopsis* (Kraepiel et al., 2011), and tobacco (Sobiczewski et al., 2017), resulting in brownish soft necrotic lesions. The lesions, however, should not be used as the sole proxy of resistance; pathogen titer should be added as another criterion, as shown in this study. In *Phalaenopsis*, lesions are smaller upon OMF colonization (Wu et al., 2011). In this study, the lesions were visually larger upon priming, but *D. fangzhongdai* titer was significantly lower. Whether these phenomena were due to the physiological difference between these orchid species is unknown. However, it is worth mentioning that leaf soft rot is usually unstoppable, causing the preventive way is by cutting the whole infected leaf because the symptom spreads quickly within days. This phenomenon is commonly observed in cultivation of various orchid species (Chi et al., 2022). Regardless, in this study, disease resistance against necrotrophic pathogens were systemically induced in orchids colonized by mycorrhizal fungi.

We investigated the potential similarity in ISR between orchids and AM plants through comparative transcriptomics. Upon root colonization by mycorrhizal fungi, the host plants alter various metabolic pathways (Fontana et al., 2009; Jacott et al., 2017; Goddard et al., 2021; Orine et al., 2022; Li et al., 2023); host plants increase their primary metabolic activity (nutrient uptake and remobilization) and temporarily lower the expression of defense-related genes (Schoenherr et al., 2019; Jing et al., 2022). In AM plants such as rice, genes associated with phosphate metabolism and phospholipid biosynthesis are upregulated, whereas non-phosphate lipids, which are later used in jasmonate biosynthesis, are downregulated (Campo and San Segundo, 2020). In *Bletilla*, the annotations related to phosphate metabolism are insufficient to conclude whether phosphate metabolism is also aligned with that of AM plants. Although it is unknown whether *Bletilla* does not prioritize phosphate metabolism or its phosphate-related genes are not differentially expressed, most studies on nutrient acquisition through OM show that orchids primarily focus on carbon and nitrogen from OMF (Stöckel et al., 2014; Fochi et al., 2016; Dearnaley and Cameron, 2017; Zahn et al., 2023). Inorganic phosphate is also taken up through OMF hyphae (Cameron et al., 2007; Davis et al., 2022) and its content is determined by trophic mode (Minasiewicz et al., 2023): mycoheterotrophic species have the highest phosphorus content, followed by partial mycoheterotrophic and autotrophic ones. Assuming that *B. striata* seedlings used in this study were already fully autotrophic, it is possible that most phosphate metabolism–related genes were not dominantly expressed even after plant colonization by OMF, unlike in AM plants.

On the other hand, after infection, the defense response was stronger after priming than before it. This is often indicated by an increase in defense-related gene expression and content of phytohormones, notably jasmonate and ethylene, which are involved in ISR and play a major role in defense against necrotrophic pathogens (Shoresh et al., 2005; Wasternack and Feussner, 2018). Mycorrhiza-colonized plants typically prioritize growth and thus allocate less resources to defense responses, including jasmonate biosynthesis (Huot et al., 2014). Once the leaf is attacked by a pathogen, jasmonate is readily increased for defense (Galis et al., 2009). Again, our comparative transcriptome analysis was still too inconclusive due to the low number of genes matched with those of rice. However, it was hinted that the expression pattern could be similar since the genes involved in the first steps of jasmonate/ethylene biosynthesis were also downregulated during priming and then upregulated after *D. fangzhongdai* infection onset. In turn, *D. fangzhongdai* titer was reduced, similar to that in other pathogen infections of AM plants. Our transcriptional evidence further supports the presence of defense priming in orchids.

In summary, our study demonstrated that the association between orchid and mycorrhizal fungi leads to ISR, thus providing a new clue for commonalities between AM and OM. As in AM plants, OMF compatibility also reflects the best colonization. The presence of a particular OMF in a substrate is a key factor in orchid seedling establishment (Gowland et al., 2013; Pujasatria et al., 2022) to provide the protocorms with nutrients and to protect against stress. We also propose that the ISR in *B. striata* involves a complex mechanism in which regulation of metabolites, defense responses, photosynthesis, and oxidative stress responses is synchronized to reduce *D. fangzhongdai* proliferation during leaf soft rot onset. Most AM studies have focused on leaves (Fujita et al., 2022; Wang et al., 2022b), but priming could be more variable in orchids than in other plants because orchid diseases also occur on pseudobulbs, rhizomes, and even flowers (Ito and Aragaki, 1977; Swett and Uchida, 2015; Li et al., 2022). Thus, the priming effects in orchids are still largely unknown if one takes into account pathogen trophic modes, latency, and the attacked organs. Further studies are needed to clarify how the defense responses were activated at the molecular level, allowing to reveal more commonalities among AM plants and orchids as well as various types of pathogens.

## Data availability statement

The datasets presented in this study can be found in online repositories. The names of the repository/repositories and accession number(s) can be found in the article/**Supplementary Material**.

## Author contributions

GP: Conceptualization, Data curation, Formal analysis, Investigation, Methodology, Visualization, Writing – original draft, Writing – review & editing. CM: Conceptualization, Data curation, Methodology, Supervision, Writing – original draft, Writing – review & editing. KY: Data curation, Resources, Writing – review & editing. SS: Data curation, Resources, Writing – review & editing. HK: Conceptualization, Project administration, Supervision, Writing – original draft, Writing – review & editing.

## References

[B1] Alexa A. Rahnenfuhrer J. (2023). topGO: Enrichment Analysis for Gene Ontology. Available online at: https://bioconductor.org/packages/topGO.

[B2] Alič Š. Pédron J. Dreo T. Van Gijsegem F. (2019). Genomic characterisation of the new *Dickeya fangzhongdai* species regrouping plant pathogens and environmental isolates. BMC Genom. 20, 1–18. doi: 10.1186/s12864-018-5332-3 PMC6329079 30634913

[B3] Bartoń K. (2023). MuMIn: Multi-model inference. R Package Version 1.47.5

[B4] Bidartondo M. I. Burghardt B. Gebauer G. Bruns T. D. Read D. J. (2004). Changing partners in the dark: isotopic and molecular evidence of ectomycorrhizal liaisons between forest orchids and trees. Proc. R. Soc Lond. B. Biol. Sci. 271, 1799–1806. doi: 10.1098/rspb.2004.2807 PMC1691795 15315895

[B5] Brundrett M. C. Tedersoo L. (2018). Evolutionary history of mycorrhizal symbioses and global host plant diversity. New Phytol. 220, 1108–1115. doi: 10.1111/nph.14976 29355963

[B6] Cameron D. D. Johnson I. Leake J. R. Read D. J. (2007). Mycorrhizal acquisition of inorganic phosphorus by the green-leaved terrestrial orchid *Goodyera repens* . Ann. Bot. 99, 831–834. doi: 10.1093/aob/mcm018 17339276 PMC2802910

[B7] Cameron D. D. Johnson I. Read D. J. Leake J. R. (2008). Giving and receiving: Measuring the carbon cost of mycorrhizas in the green orchid, *Goodyera repens* . New Phytol. 180, 176–184. doi: 10.1111/j.1469-8137.2008.02533.x 18627489

[B8] Campo S. San Segundo B. (2020). Systemic induction of phosphatidylinositol-based signaling in leaves of arbuscular mycorrhizal rice plants. Sci. Rep. 10, 1–17. doi: 10.1038/s41598-020-72985-6 32985595 PMC7522983

[B9] Cating R. A. Palmateer A. J. (2011). Bacterial Soft Rot of *Oncidium* Orchids Caused by a *Dickeya* sp. (*Pectobacterium chrysanthemi*) in Florida. Plant Dis. 95, 74. doi: 10.1094/PDIS-07-10-0523 30743689

[B10] Chen S. Zhou Y. Chen Y. Gu J. Gu Z. Eils R.. (2018). Fastp: An ultra-fast all-in-one FASTQ preprocessor. Bioinformatics 34, 2847–2849. doi: 10.1093/bioinformatics/bty560 PMC6129281 30423086

[B11] Chi N. M. Anh D. T. K. Hung T. X. Nhung N. P. Bao H. Q. Toan D. V.. (2022). Soft rot caused by *Dickeya fangzhongdai* in epiphytic orchids in Vietnam. Can. J. Plant Path. 44, 386–399. doi: 10.1080/07060661.2021.1998226

[B12] Chomicki G. Bidel L. P. R. Ming F. Coiro M. Zhang X. Wang Y.. (2015). The velamen protects photosynthetic orchid roots against UV-B damage, and a large dated phylogeny implies multiple gains and losses of this function during the Cenozoic. New Phytol. 205, 1330–1341. doi: 10.1111/nph.13106 25345817

[B13] Cope K. R. Kafle A. Yakha J. K. Pfeffer P. E. Strahan G. D. Garcia K.. (2022). Physiological and transcriptomic response of *Medicago truncatula* to colonization by high- or low-benefit arbuscular mycorrhizal fungi. Mycorrhiza 32, 281–303. doi: 10.1007/s00572-022-01077-2 35511363

[B14] Cosentino S. Iwasaki W. (2019). SonicParanoid: Fast, accurate and easy orthology inference. Bioinformatics 35, 149–151. doi: 10.1093/bioinformatics/bty631 30032301 PMC6298048

[B15] David L. Harmon A. C. Chen S. (2019). Plant immune responses - from guard cells and local responses to systemic defense against bacterial pathogens. Plant Signal Behav. 14, 1–9. doi: 10.1080/15592324.2019.1588667 PMC6512940 30907231

[B16] Davis B. Lim W. H. Lambers H. Dixon K. W. Read D. J. (2022). Inorganic phosphorus nutrition in green-leaved terrestrial orchid seedlings. Ann. Bot. 129, 669–678. doi: 10.1093/aob/mcac030 35247265 PMC9113155

[B17] Dearnaley J. D. W. Cameron D. D. (2017). Nitrogen transport in the orchid mycorrhizal symbiosis – further evidence for a mutualistic association. New Phytol. 213, 10–12. doi: 10.1111/nph.14357 27891646

[B18] Dearnaley J. D. W. Martos F. Selosse M. A. (2012). “Orchid mycorrhizas: Molecular ecology, physiology, evolution and conservation aspects,” in Fungal Associations. Ed. Hock B. (Springer Berlin, Heidelberg), 207–230. doi: 10.1007/978-3-642-30826-0_12

[B19] Dreischhoff S. Das I. S. Jakobi M. Kasper K. Polle A. (2020). Local responses and systemic induced resistance mediated by ectomycorrhizal fungi. Front. Plant Sci. 11. doi: 10.3389/fpls.2020.590063 PMC7767828 33381131

[B20] Eck J. L. Kytöviita M. M. Laine A. L. (2022). Arbuscular mycorrhizal fungi influence host infection during epidemics in a wild plant pathosystem. New Phytol. 236, 1922–1935. doi: 10.1111/nph.18481 36093733 PMC9827988

[B21] Fochi V. Chitarra W. Kohler A. Voyron S. Singan V. R. Lindquist E. A.. (2016). Fungal and plant gene expression in the *Tulasnella calospora - Serapias vomeracea* symbiosis provides clues about nitrogen pathways in orchid mycorrhizas. New Phytol. 213, 365–379. doi: 10.1111/nph.14279 27859287

[B22] Fogell D. J. Kundu S. Roberts D. L. (2019). Genetic homogenisation of two major orchid viruses through global trade-based dispersal of their hosts. Plants People Planet 1, 356–362. doi: 10.1002/ppp3.46

[B23] Fontana A. Reichelt M. Hempel S. Gershenzon J. Unsicker S. B. (2009). The effects of arbuscular mycorrhizal fungi on direct and indirect defense metabolites of *Plantago lanceolata* L. J. Chem. Ecol. 35, 833–843. doi: 10.1007/s10886-009-9654-0 19568812 PMC2712616

[B24] Fracchia S. Aranda-Rickert A. Rothen C. Sede S. (2016). Associated fungi, symbiotic germination and *in vitro* seedling development of the rare Andean terrestrial orchid. Chloraea riojana. Flora 224, 106–111. doi: 10.1016/j.flora.2016.07.008

[B25] Fu S. F. Tsai T. M. Chen Y. R. Liu C. P. Haiso L. J. Syue L. H.. (2012). Characterization of the early response of the orchid, *Phalaenopsis amabilis*, to *Erwinia chrysanthemi* infection using expression profiling. Physiol. Plant 145, 406–425. doi: 10.1111/j.1399-3054.2012.01582.x 22268629

[B26] Fuji M. Miura C. Yamamoto T. Komiyama S. Suetsugu K. Yagame T.. (2020). Relative effectiveness of *Tulasnella* fungal strains in orchid mycorrhizal symbioses between germination and subsequent seedling growth. Symbiosis 81, 53–63. doi: 10.1007/s13199-020-00681-0

[B27] Fujita M. Kusajima M. Fukagawa M. Okumura Y. Nakajima M. Akiyama K.. (2022). Response of tomatoes primed by mycorrhizal colonization to virulent and avirulent bacterial pathogens. Sci. Rep. 12, 1–12. doi: 10.1038/s41598-022-08395-7 35304874 PMC8933586

[B28] Galis I. Gaquerel E. Pandey S. P. Baldwin I. T. (2009). Molecular mechanisms underlying plant memory in JA-mediated defence responses. Plant Cell Environ. 32, 617–627. doi: 10.1111/j.1365-3040.2008.01862.x 18657055

[B29] Girlanda M. Segreto R. Cafasso D. Liebel H. T. Rodda M. Ercole E.. (2011). Photosynthetic Mediterranean meadow orchids feature partial mycoheterotrophy and specific mycorrhizal associations1. Am. J. Bot. 98, 1148–1163. doi: 10.3732/ajb.1000486 21712419

[B30] Goddard M. L. Belval L. Martin I. R. Roth L. Laloue H. Deglène-Benbrahim L.. (2021). Arbuscular mycorrhizal symbiosis triggers major changes in primary metabolism together with modification of defense responses and signaling in both roots and leaves of Vitis vinifera. Front. Plant Sci. 12. doi: 10.3389/fpls.2021.721614 PMC8424087 34512700

[B31] Gowland K. M. van der Merwe M. M. Linde C. C. Clements M. A. Nicotra A. B. (2013). The host bias of three epiphytic Aeridinae orchid species is reflected, but not explained, by mycorrhizal fungal associations. Am. J. Bot. 100, 764–777. doi: 10.3732/ajb.1200411 23545217

[B32] Gu Z. Eils R. Schlesner M. (2016). Complex heatmaps reveal patterns and correlations in multidimensional genomic data. Bioinformatics 32, 2847–2849. doi: 10.1093/bioinformatics/btw313 27207943

[B33] Guigard L. Jobert L. Busset N. Moulin L. Czernic P. (2023). Symbiotic compatibility between rice cultivars and arbuscular mycorrhizal fungi genotypes affects rice growth and mycorrhiza-induced resistance. Front. Plant Sci. 14. doi: 10.3389/fpls.2023.1278990 PMC10628536 37941658

[B34] Haney C. H. Wiesmann C. L. Shapiro L. R. Melnyk R. A. O’Sullivan L. R. Khorasani S.. (2018). Rhizosphere-associated *Pseudomonas* induce systemic resistance to herbivores at the cost of susceptibility to bacterial pathogens. Mol. Ecol. 27, 1833–1847. doi: 10.1111/mec.14400 29087012

[B35] Hélias V. Hamon P. Huchet E. Wolf J. Andrivon D. (2012). Two new effective semiselective crystal violet pectate media for isolation of Pectobacterium and *Dickeya* . Plant Pathol. 61, 339–345. doi: 10.1111/j.1365-3059.2011.02508.x

[B36] Huot B. Yao J. Montgomery B. L. He S. Y. (2014). Growth-defense tradeoffs in plants: A balancing act to optimize fitness. Mol. Plant 7, 1267–1287. doi: 10.1093/mp/ssu049 24777989 PMC4168297

[B37] Ito J. S. Aragaki M. (1977). Botrytis blossom blight of *Dendrobium* . Phytopathology 67, 820–824. doi: 10.1094/Phyto-67-820

[B38] Jacott C. N. Murray J. D. Ridout C. J. (2017). Trade-offs in arbuscular mycorrhizal symbiosis: Disease resistance, growth responses and perspectives for crop breeding. Agronomy 7, 1–18. doi: 10.3390/agronomy7040075

[B39] Jing S. Li Y. Zhu L. Su J. Yang T. Liu B.. (2022). Transcriptomics and metabolomics reveal effect of arbuscular mycorrhizal fungi on growth and development of apple plants. Front. Plant Sci. 13. doi: 10.3389/fpls.2022.1052464 PMC9641280 36388499

[B40] Joko T. Subandi A. Kusumandari N. Wibowo A. Priyatmojo A. (2014). Activities of plant cell wall-degrading enzymes by bacterial soft rot of orchid. Arch. Phytopathol. Plant Prot. 47, 1239–1250. doi: 10.1080/03235408.2013.838374

[B41] Kakouridis A. Hagen J. A. Kan M. P. Mambelli S. Feldman L. J. Herman D. J.. (2022). Routes to roots: direct evidence of water transport by arbuscular mycorrhizal fungi to host plants. New Phytol. 236, 210–221. doi: 10.1111/nph.18281 35633108 PMC9543596

[B42] Kawahara Y. de la Bastide M. Hamilton P. Kanamori H. McCombie W. R. Ouyang S. (2013). Improvement of the *Oryza sativa* Nipponbare reference genome using next generation sequence and optical map data. Rice. 6, 1–10. doi: 10.1186/1939-8433-6-4 PMC5395016 24280374

[B43] Keith L. M. Sewake K. T. Zee F. T. (2005). Isolation and characterization of *Burkholderia gladioli* from orchids in Hawaii. Plant Dis. 89, 1273–1278. doi: 10.1094/PD-89-1273 30791304

[B44] Khamtham J. Akarapisan A. (2019). *Acidovorax avenae subsp. cattleyae* causes bacterial brown spot disease on terrestrial orchid *Habenaria lindleyana* in Thailand. J. Plant Pathol. 101, 31–37. doi: 10.1007/s42161-018-0135-6

[B45] Kraepiel Y. Pédron J. Patrit O. Simond-Côte E. Hermand V. van Gijsegem F. (2011). Analysis of the plant Bos1 mutant highlights necrosis as an efficient defence mechanism during *D. dadantii/Arabidopsis thaliana* interaction. PloS One 6, 1–10. doi: 10.1371/journal.pone.0018991 PMC3080887 21533045

[B46] Kuga Y. Sakamoto N. Yurimoto H. (2014). Stable isotope cellular imaging reveals that both live and degenerating fungal pelotons transfer carbon and nitrogen to orchid protocorms. New Phytol. 202, 594–605. doi: 10.1111/nph.12700 24494717

[B47] Kuznetsova A. Brockhoff P. B. Christensen R. H. B. (2017). lmerTest Package: Tests in Linear Mixed Effects Models. J Stat Softw. 82: 1–26. doi: 10.18637/jss.v082.i13

[B48] Langmead B. Salzberg S. L. (2012). Fast gapped-read alignment with Bowtie 2. Nat. Method. 9, 357–359. doi: 10.1038/nmeth.1923 PMC3322381 22388286

[B49] Lee Y. A. Yu C. P. (2006). A differential medium for the isolation and rapid identification of a plant soft rot pathogen, *Erwinia chrysanthemi* . J. Microbiol. Method. 64, 200–206. doi: 10.1016/j.mimet.2005.04.031 15927293

[B50] Li M. Kim C. (2022). Chloroplast ROS and stress signaling. Plant Commun. 3, 100264. doi: 10.1016/j.xplc.2021.100264 35059631 PMC8760138

[B51] Li Y. Nan Z. Matthew C. Wang Y. Duan T. (2023). Arbuscular mycorrhizal fungus changes alfalfa (*Medicago sativa*) metabolites in response to leaf spot (*Phoma medicaginis*) infection, with subsequent effects on pea aphid (*Acyrthosiphon pisum*) behavior. New Phytol. 239, 286–300. doi: 10.1111/nph.18924 37010085

[B52] Li J. Zhang M. Yang Z. Li C. (2022). *Botrytis cinerea* causes flower gray mold in *Gastrodia elata* in China. Crop Prot. 155, 1–4. doi: 10.1016/j.cropro.2022.105923

[B53] Liu J. Maldonado-Mendoza I. Lopez-Meyer M. Cheung F. Town C. D. Harrison M. J. (2007). Arbuscular mycorrhizal symbiosis is accompanied by local and systemic alterations in gene expression and an increase in disease resistance in the shoots. Plant J. 50, 529–544. doi: 10.1111/j.1365-313X.2007.03069.x 17419842

[B54] Lopes U. P. Zambolim L. Pereira O. L. (2009). First report of *Lasiodiplodia theobromae* causing leaf blight on the orchid *Catasetum fimbriatum* in Brazil. *Australas* . Plant Dis. Note. 4, 64–65. doi: 10.1071/DN09027

[B55] Lu Y. Yao J. (2018). Chloroplasts at the crossroad of photosynthesis, pathogen infection and plant defense. Int. J. Mol. Sci. 19, 1–37. doi: 10.3390/ijms19123900 PMC6321325 30563149

[B56] Marquez N. Giachero M. L. Gallou A. Debat H. J. Cranenbrouck S. di Rienzo J. A.. (2018). Transcriptional changes in mycorrhizal and nonmycorrhizal soybean plants upon infection with the fungal pathogen *Macrophomina phaseolina* . Mol. Plant-Microbe Interact. 31, 842–855. doi: 10.1094/MPMI-11-17-0282-R 29498566

[B57] McKendrick S. L. Leake J. R. Lee Taylor D. Read D. J. (2004). Symbiotic germination and development of the myco-heterotrophic orchid *Neottia nidus-avis* in nature and its requirement for locally distributed *Sebacina* spp. New Phytol. 163, 405–423. doi: 10.1111/j.1469-8137.2004.01115.x 33873615

[B58] Meng Y. Y. Zhang W. L. Selosse M. A. Gao J. Y. (2019). Are fungi from adult orchid roots the best symbionts at germination? A case study. Mycorrhiza 29, 541–547. doi: 10.1007/s00572-019-00907-0 31312918

[B59] Minasiewicz J. Zwolicki A. Figura T. Novotná A. Bocayuva M. F. Jersáková J.. (2023). Stoichiometry of carbon, nitrogen and phosphorus is closely linked to trophic modes in orchids. BMC Plant Biol. 23, 1–11. doi: 10.1186/s12870-023-04436-z 37700257 PMC10496321

[B60] Miura C. Furui Y. Yamamoto T. Kanno Y. Honjo M. Yamaguchi K.. (2024). Autoactivation of mycorrhizal symbiosis signaling through gibberellin deactivation in orchid seed germination. Plant Physiol. 194, 1–18. doi: 10.1093/plphys/kiad517 PMC10756758 37776523

[B61] Miura C. Saisho M. Yagame T. Yamato M. Kaminaka H. (2019). *Bletilla striata* (Orchidaceae) seed coat restricts the invasion of fungal hyphae at the initial stage of fungal colonization. Plants 8, 1–11. doi: 10.3390/plants8080280 PMC6724134 31405202

[B62] Miura C. Yamaguchi K. Miyahara R. Yamamoto T. Fuji M. Yagame T.. (2018). The mycoheterotrophic symbiosis between orchids and mycorrhizal fungi possesses major components shared with mutualistic plant-mycorrhizal symbioses. Mol. Plant Microbe Interact. 31, 1032–1047. doi: 10.1094/MPMI-01-18-0029-R 29649962

[B63] Orine D. Defossez E. Vergara F. Uthe H. van Dam N. M. Rasmann S. (2022). Arbuscular mycorrhizal fungi prevent the negative effect of drought and modulate the growth-defence trade-off in tomato plants. J. Sustain. Agric. Environ. 1, 177–190. doi: 10.1002/sae2.12018

[B64] Otero J. T. Bayman P. Ackerman J. D. (2005). Variation in mycorrhizal performance in the epiphytic orchid *Tolumnia variegata in vitro*: The potential for natural selection. Evol. Ecol. 19, 29–43. doi: 10.1007/s10682-004-5441-0

[B65] Parniske M. (2008). Arbuscular mycorrhiza: the mother of plant root endosymbioses. Nat. Rev. Microbiol. 6, 763–775. doi: 10.1038/nrmicro1987 18794914

[B66] Pérez F. J. Rubio S. (2006). An improved chemiluminescence method for hydrogen peroxide determination in plant tissues. Plant Growth Regul. 48, 89–95. doi: 10.1007/s10725-005-5089-y

[B67] Peterson R. L. Massicote H. B. Melville L. H. (2004). Mycorrhizas: Anatomy and Cell Biology (Ottawa: NRC Research Press). doi: 10.1017/s0269-915x(05)21306-x

[B68] Pieterse C. M. J. Zamioudis C. Berendsen R. L. Weller D. M. Van Wees S. C. M. Bakker P. A. H. M. (2014). Induced systemic resistance by beneficial microbes. Annu. Rev. Phytopathol. 52, 347–375. doi: 10.1146/annurev-phyto-082712-102340 24906124

[B69] Pujasatria G. C. Nishiguchi I. Miura C. Yamato M. Kaminaka H. (2022). Orchid mycorrhizal fungi and ascomycetous fungi in epiphytic *Vanda falcata* roots occupy different niches during growth and development. Mycorrhiza 32, 481–495. doi: 10.1007/s00572-022-01089-y 35844010

[B70] Qin J. Zhang W. Ge Z. W. Zhang S. B. (2019). Molecular identifications uncover diverse fungal symbionts of *Pleione* (Orchidaceae). Fungal Ecol. 37, 19–29. doi: 10.1016/j.funeco.2018.10.003

[B71] Ross A. F. (1966). “Systemic effects of local lesion formation,” in Viruses of Plants: Their Isolation, Purification, and Characterization: the Mechanism of Plant Virus Infection, Synthesis of Viral Protein and Viral Nucleic Acid, and Plant Reactions Evoked by Viruses. Eds. Beemster A. B. R. Dijkstra J. (North-Holland Publishing Company, Wageningen), 127–150.

[B72] Säle V. Palenzuela J. Azcón-Aguilar C. Sánchez-Castro I. da Silva G. A. Seitz B.. (2021). Ancient lineages of arbuscular mycorrhizal fungi provide little plant benefit. Mycorrhiza 31, 559–576. doi: 10.1007/s00572-021-01042-5 34327560 PMC8484173

[B73] Samson R. Legendre J. B. Christen R. Fischer-Le Saux M. Achouak W. Gardan L. (2005). Transfer of *Pectobacterium chrysanthemi* (Burkholder et al. 1953) Brenner et al. 1973 and *Brenneria paradisiaca* to the genus *Dickeya* gen. nov. as *Dickeya chrysanthemi* comb. nov. and *Dickeya paradisiaca* comb. nov. and delineation of four novel species, Dick. Int. J. Syst. Evol. Microbiol. 55, 1415–1427. doi: 10.1099/ijs.0.02791-0 16014461

[B74] Sathiyadash K. Muthukumar T. Murugan S. B. Sathishkumar R. Pandey R. R. (2014). *In vitro* symbiotic seed germination of South Indian endemic orchid *Coelogyne nervosa* . Mycoscience 55, 183–189. doi: 10.1016/j.myc.2013.08.005

[B75] Schoenherr A. P. Rizzo E. Jackson N. Manosalva P. Gomez S. K. (2019). Mycorrhiza-induced resistance in potato involves priming of defense responses against cabbage looper (Noctuidae: Lepidoptera). Environ. Entomol. 48, 370–381. doi: 10.1093/ee/nvy195 30715218

[B76] Selosse M. A. Minasiewicz J. Boullard B. (2017). An annotated translation of Noël Bernard’s 1899 article ‘On the germination of *Neottia nidus-avis.*’. Mycorrhiza 27, 611–618. doi: 10.1007/s00572-017-0774-z 28421312

[B77] Shoresh M. Yedidia I. Chet I. (2005). Involvement of jasmonic acid/ethylene signaling pathway in the systemic resistance induced in cucumber by *Trichoderma asperellum* T203. Phytopathology 95, 76–84. doi: 10.1094/PHYTO-95-0076 18943839

[B78] Silva M. Pereira O. L. (2007). First report of *Guignardia endophyllicola* leaf blight on *Cymbidium* (Orchidaceae) in Brazil. Australas. Plant Dis. Note. 2, 31–32. doi: 10.1071/DN07015

[B79] Smith S. E. Read D. (2008). Mycorrhizal Symbiosis. 3rd ed. (New York: Academic Press Inc).

[B80] Smyth G. K. Ritchie M. E. Law C. W. Alhamdoosh M. Su S. Dong X.. (2018). RNA-seq analysis is easy as 1-2-3 with limma, Glimma and edgeR. F1000Res 5, 1–30. doi: 10.12688/f1000research.9005.3 PMC4937821 27441086

[B81] Sobiczewski P. Iakimova E. T. Mikiciński A. Węgrzynowicz-Lesiak E. Dyki B. (2017). Necrotrophic behaviour of *Erwinia amylovora* in apple and tobacco leaf tissue. Plant Pathol. 66, 842–855. doi: 10.1111/ppa.12631

[B82] Srivastava S. Kadooka C. Uchida J. Y. (2018). *Fusarium* species as pathogen on orchids. Microbiol. Res. 207, 188–195. doi: 10.1016/j.micres.2017.12.002 29458853

[B83] Stöckel M. Těšitelová T. Jersáková J. Bidartondo M. I. Gebauer G. (2014). Carbon and nitrogen gain during the growth of orchid seedlings in nature. New Phytol. 202, 606–615. doi: 10.1111/nph.12688 24444001

[B84] Strullu-Derrien C. Selosse M. A. Kenrick P. Martin F. M. (2018). The origin and evolution of mycorrhizal symbioses: from palaeomycology to phylogenomics. New Phytol. 220, 1012–1030. doi: 10.1111/nph.15076 29573278

[B85] Suharjo R. Sawada H. Takikawa Y. (2014). Phylogenetic study of Japanese *Dickeya* spp. and development of new rapid identification methods using PCR-RFLP. J. Gen. Plant Pathol. 80, 237–254. doi: 10.1007/s10327-014-0511-9

[B86] Suwannarach N. Kumla J. Lumyong S. (2018). Leaf spot on *Cattleya* orchid caused by *Neoscytalidium orchidacearum* in Thailand. Can. J. Plant Pathol. 40, 109–114. doi: 10.1080/07060661.2017.1414882

[B87] Swett C. S. Uchida J. Y. (2015). Characterization of *Fusarium* diseases on commercially grown orchids in Hawaii. Plant Pathol. 64, 648–654. doi: 10.1111/ppa.12290

[B88] Tsai C. F. Huang C. H. Wu F. H. Lin C. H. Lee C. H. Yu S. S.. (2022). Intelligent image analysis recognizes important orchid viral diseases. Front. Plant Sci. 13. doi: 10.3389/fpls.2022.1051348 PMC9755359 36531380

[B89] Umata H. Ota Y. Yamada M. Watanabe Y. Gale S. W. (2013). Germination of the fully myco-heterotrophic orchid *Cyrtosia septentrionalis* is characterized by low fungal specificity and does not require direct seed-mycobiont contact. Mycoscience 54, 343–352. doi: 10.1016/j.myc.2012.12.003

[B90] van Peer R. Niemann G. J. Schippers B. (1990). Induced resistance and phytoalexin accumulation in biological control of fusarium wilt of carnation by *pseudomonas* sp. Strain WCS417r. Phytopathology 81, 728–734. doi: 10.1094/Phyto-81-728

[B91] Vierheilig H. Coughlan A. P. Wyss U. Piche Y. (1998). Ink and vinegar, a simple staining technique for arbuscular-mycorrhizal fungi. Appl. Environ. Microbiol. 64, 5004–5007. doi: 10.1128/AEM.64.12.5004-5007.1998 9835596 PMC90956

[B92] Vlot A. C. Sales J. H. Lenk M. Bauer K. Brambilla A. Sommer A.. (2021). Systemic propagation of immunity in plants. New Phytol. 229, 1234–1250. doi: 10.1111/nph.16953 32978988

[B93] Wang D. Gebauer G. Jacquemyn H. Zahn F. E. Gomes S. I. F. Lorenz J.. (2023). Variation in mycorrhizal communities and the level of mycoheterotrophy in grassland and Forest populations of *Neottia ovata* (Orchidaceae). Funct. Ecol. 37, 1948–1961. doi: 10.1111/1365-2435.14354

[B94] Wang H. Hao Z. Zhang X. Xie W. Chen B. (2022a). Arbuscular mycorrhizal fungi induced plant resistance against fusarium wilt in jasmonate biosynthesis defective mutant and wild type of tomato. J. Fungi 8, 1–14. doi: 10.3390/jof8050422 PMC9146357 35628678

[B95] Wang M. Tang W. Xiang L. Chen X. Shen X. Yin C.. (2022b). Involvement of *MdWRKY40* in the defense of mycorrhizal apple against *Fusarium solani* . BMC Plant Biol. 22, 1–15. doi: 10.1186/s12870-022-03753-z 35918651 PMC9344649

[B96] Wasternack C. Feussner I. (2018). The oxylipin pathways: biochemistry and function. Annu. Rev. Plant Biol. 69, 363–386. doi: 10.1146/annurev-arplant-042817-040440 29166128

[B97] Wei X. Y. Deng W. L. Chu C. C. (2021). Phylogenetic and phenotypic analyses on *Dickeya* spp. isolated from different host plants in Taiwan. J. Phytopathol. 169, 678–691. doi: 10.1111/jph.13038

[B98] Wei G. Kloepper J. W. Tuzun S. (1991). Induction of systemic resistance of cucumber to *colletotrichum orbiculare* by select strains of plant growth-promoting rhizobacteria. Phytopathology 81, 1508–1512. doi: 10.1094/Phyto-81-1508

[B99] Wellburn A. R. (1994). The Spectral Determination of Chlorophylls a and b, as well as Total Carotenoids, Using Various Solvents with Spectrophotometers of Different Resolution. J. Plant Physiol. 144, 307–313. doi: 10.1016/S0176-1617(11)81192-2

[B100] Wu P. H. Huang D. D. Chang D. C. N. (2011). Mycorrhizal symbiosis enhances *Phalaenopsis* orchid’s growth and resistance to *Erwinia chrysanthemi* . Afr. J. Biotechnol. 10, 10095–10100. doi: 10.5897/AJB11.1310

[B101] Xing X. Jacquemyn H. Gai X. Gao Y. Liu Q. Zhao Z.. (2019). The impact of life form on the architecture of orchid mycorrhizal networks in tropical forest. Oikos 128, 1254–1264. doi: 10.1111/oik.06363

[B102] Yamamoto T. Miura C. Fuji M. Nagata S. Otani Y. Yagame T.. (2017). Quantitative evaluation of protocorm growth and fungal colonization in *Bletilla striata* (Orchidaceae) reveals less-productive symbiosis with a non-native symbiotic fungus. BMC Plant Biol. 17, 1–10. doi: 10.1186/s12870-017-1002-x 28222700 PMC5320772

[B103] Ye W. Jiang J. Lin Y. Yeh K. W. Lai Z. Xu X.. (2019). Colonisation of Oncidium orchid roots by the endophyte *Piriformospora indica* restricts *Erwinia chrysanthemi* infection, stimulates accumulation of NBS-LRR resistance gene transcripts and represses their targeting micro-RNAs in leaves. BMC Plant Biol. 19, 1–16. doi: 10.1186/s12870-019-2105-3 31888486 PMC6937650

[B104] Yukawa T. Ogura-Tsujita Y. Shefferson R. P. Yokoyama J. (2009). Mycorrhizal diversity in *Apostasia* (Orchidaceae) indicates the origin and evolution of orchid mycorrhiza. Am. J. Bot. 96, 1997–2009. doi: 10.3732/ajb.0900101 21622320

[B105] Zahn F. E. Söll E. Chapin T. K. Wang D. Gomes S. I. F. Hynson N. A.. (2023). Novel insights into orchid mycorrhiza functioning from stable isotope signatures of fungal pelotons. New Phytol. 239, 1449–1463. doi: 10.1111/nph.18991 37343598

[B106] Zettler L. W. Stewart S. L. Bowles M. L. Jacobs K. A. (2001). Mycorrhizal fungi and cold-assisted symbiotic germination of the federally threatened eastern prairie fringed orchid, *platanthera leucophaea* (Nuttall) lindley. Am. Midl. Nat. 145, 168–175. doi: 10.1674/0003-0031(2001)145[0168:mfacas]2.0.co;2

[B107] Zhou A. Nie J. Tian Y. Chuan J. Hu B. Zou J.. (2021). First report of *dickeya fangzhongdai* causing soft rot in orchid in Canada. Plant Dis. 105, 4149. doi: 10.1094/PDIS-04-21-0771-PDN

[B108] Zhu Z. (2014). Molecular basis for jasmonate and ethylene signal interactions in Arabidopsis. J. Exp. Bot. 65, 5743–5748. doi: 10.1093/jxb/eru349 25165148

